# Impact of the GeneXpert^®^ MTB/RIF rapid molecular test on tuberculosis detection: temporal trends and vulnerable territories*

**DOI:** 10.1590/1518.8345.4412.3441

**Published:** 2021-07-19

**Authors:** Thaís Zamboni Berra, Alexandre Tadashi Inomata Bruce, Yan Mathias Alves, Antônio Carlos Vieira Ramos, Clóvis Luciano Giacomet, Ricardo Alexandre Arcêncio

**Affiliations:** 1 Universidade de São Paulo, Escola de Enfermagem de Ribeirão Preto, PAHO/WHO Collaborating Centre for Nursing Research Development, Ribeirão Preto, SP, Brazil.; 2 Scholarship holder at the Fundação de Amparo à Pesquisa do Estado de São Paulo (FAPESP), Brazil.; 3 Scholarship holder at the Conselho Nacional de Desenvolvimento Científico e Tecnológico (CNPq), Brazil.; 4 Scholarship holder at the Coordenação de Aperfeiçoamento de Pessoal de Nível Superior (CAPES), Brazil.

**Keywords:** Tuberculosis, Mycobacterium tuberculosis, Diagnosis, Incidence, Time Series Studies, Spatial Analysis, Tuberculose, Mycobacterium tuberculosis, Diagnóstico, Incidência, Estudos de Série Temporal, Análise Espacial, Tuberculosis, Mycobacterium tuberculosis, Diagnostico, Incidencia, Estudios de Series Temporales, Análisis Espacial

## Abstract

**Objective:**

to assess the impact of the GeneXpert^®^ MTB/RIF rapid molecular test on tuberculosis detection, to analyze the temporal trend of the event and to identify vulnerable territories in a Brazilian municipality.

**Method:**

an ecological study carried out in Ribeirão Preto, São Paulo, Brazil, a municipality considered a priority in tuberculosis control due to the high number of cases. To classify the temporal trend, the Prais-Winsten method and the Interrupted Time Series were used to identify changes in the disease incidence. Kernel intensity analysis was applied to identify vulnerable areas.

**Results:**

the temporal trend of tuberculosis decreased by 18.1%/year and by 6.9%/year for children under 15 years old. The North District decreased by 6.67%/year and the East District increased by 17.5%/year in the incidence of tuberculosis. Resistant tuberculosis, after the implementation of the Rapid Molecular Test, increased by 0.6% per year. The South and West Districts showed a higher density of cases, with a range from 45 to 79 tuberculosis cases per square kilometer (km^2^).

**Conclusion:**

although resistant tuberculosis is not a problem in the scenario, the study showed an increase in its incidence, which puts it on alert. The use of spatial analysis enabled the identification of priority areas, putting them in evidence for health surveillance actions.

## Abstract (pt)

**Objetivo:**

avaliar o impacto do teste rápido molecular GeneXpert^®^ MTB/RIF na detecção da tuberculose, analisar a tendência temporal do evento e identificar territórios vulneráveis em município brasileiro.

**Método:**

estudo ecológico realizado em Ribeirão Preto, São Paulo, Brasil, município considerado prioritário no controle da tuberculose devido ao elevado número de casos. Para classificar a tendência temporal foi utilizado o método de Prais-Winsten e a Série Temporal Interrompida para identificar mudanças na incidência da doença. Aplicou-se a análise de intensidade de Kernel para a identificação de áreas vulneráveis.

**Resultados:**

a tendência temporal da tuberculose apresentou decréscimo de 18,1%/ano e de 6,9%/ano em menores de 15 anos. O Distrito Norte apresentou decréscimo de 6,67%/ano e o Distrito Leste crescimento de 17,5%/ano, na incidência de tuberculose. A tuberculose resistente, após a implementação do teste rápido molecular, apresentou aumento de 0,6% por ano. Os Distritos Sul e Oeste apresentaram maior densidade de casos, com variação de 45 a 79 casos de tuberculose por quilômetro quadrado (km^2^).

**Conclusão:**

apesar da tuberculose resistente não ser um problema no cenário, o estudo evidenciou um crescimento na sua incidência, o que o coloca em estado de alerta. O uso da análise espacial possibilitou a identificação das áreas prioritárias, colocando-as em evidência para ações de vigilância em saúde.

## Abstract (es)

**Objetivo:**

evaluar el impacto de la prueba rápida molecular GeneXpert^®^ MTB/RIF en la detección de tuberculosis, analizar la tendencia temporal del evento e identificar territorios vulnerables en un municipio brasileño.

**Método:**

estudio ecológico realizado en Ribeirão Preto, São Paulo, Brasil, municipio considerado prioritario en el control de la tuberculosis por el elevado número de casos. Se utilizó el método de Prais-Winsten para clasificar la tendencia temporal y la técnica de Series de Tiempo Interrumpidas para identificar cambios en la incidencia de la enfermedad. Se aplicó un análisis de intensidad de Kernel para identificar áreas vulnerables.

**Resultados:**

la tendencia temporal de la tuberculosis disminuyó un 18,1%/año y un 6,9%/año en los menores de 15 años. La incidencia de tuberculosis disminuyó un 6,67%/año en el Distrito Norte y aumentó un 17,5%/año en el Distrito Este. La tuberculosis resistente, después de la implementación de la Prueba Molecular Rápida, aumentó un 0,6% anual. Los Distritos Sur y Oeste presentaron una mayor densidad de casos, con un rango de 45 a 79 casos de tuberculosis por kilómetros cuadrados (km^2^).

**Conclusión:**

aunque la tuberculosis resistente no representa un problema en el escenario, el estudio mostró un aumento en la incidencia, lo que genera una señal de alerta. El uso del análisis espacial permitió identificar áreas prioritarias, para que puedan llevarse a cabo acciones de vigilancia en salud.

## Introduction

Tuberculosis (TB), a communicable disease, is one of the top 10 causes of death worldwide and the predominant reason for death by a single infectious agent. It is estimated that, approximately, one third of the world population is infected with Mycobacterium tuberculosis and at risk of developing the disease^1^.

According to the World Health Organization, in 2018 there were approximately 10 million new cases in the world, a number that has been maintained in recent years, with 57% of these cases being men, 32% women, and 11% people under 15 years of age. In that same year, an estimated 1.2 million deaths due to tuberculosis and 251,000 deaths due to tuberculosis and Human Immunodeficiency Virus (TB-HIV) coinfection^1^.

Brazil occupies the 20th position among the 30 countries that concentrate 90% of the global burden of tuberculosis. In 2018, more than 73,000 new cases were recorded in the country, indicating a detection rate of 35.0 cases/100,000 inhabitants, 13,610 cases of tuberculosis retreatments and just over 500 new cases of drug resistance^2^.

In general, between 2009, when the incidence was 38.3 cases/100,000 inhabitants, and the year 2018, it was possible to observe a mean annual drop of nearly 1% in the tuberculosis incidence rate in the country; however, it is worth noting that the incidence of the disease increased between the years 2017 and 2018, when compared to the years 2014 to 2016^3^. The mortality rate, in the same period, was 2.2 cases/100,000 inhabitants^3^.

In view of the limitations of the conventional diagnostic tests, the Rapid Molecular Test for Tuberculosis (RMT-TB) was carried out using the GeneXpert^®^ MTB/RIF system, used for the detection of Mycobacterium tuberculosis and resistance to rifampicin^4^. This system was recommended by the World Health Organization in 2010^4^, being incorporated by various health systems and most Latin American countries for the diagnosis of tuberculosis. In Brazil, the test was approved by the National Commission for the Incorporation of Technologies (Comissão Nacional de Incorporação de Tecnologias, CONITEC - SUS) in the Unified Health System (Sistema Unico de Saúde - SUS in Portuguese language) in 2013 and the incorporation into the SUS occurred in the same year, with the acquisition of 160 pieces of equipment distributed throughout the territory^4^.

According to the World Health Organization, in samples where the sensitivity of smear microscopy was 65%, the RMT-TB showed a sensitivity of 88%, increasing by 23% the detection of tuberculosis. The World Health Organization recommends conducting operational research studies aimed at evaluating the contributions of the RMT-TB to the health systems such as expenses, impact for the patient and society^5^^-^ 7; however, no studies were found in the literature that sought to assess the impact of the RMT-TB in the detection of tuberculosis cases in normal circumstances, of health service activities.

The study aims to assess the impact of the GeneXpert^®^ MTB/RIF rapid molecular test on tuberculosis detection, to analyze the temporal trend of the event and to identify vulnerable territories in a Brazilian municipality.

## Method

An ecological study^8^ carried out in Ribeirão Preto, located 314 kilometers (km) from the capital of the State of São Paulo, Brazil, which has an approximate area of 650 km^2^ and a demographic density of 995.3 inhabitants/km^2^ 9. It is noteworthy that this city was selected as the setting for the study because it is considered one of the priority municipalities, due to the high number of tuberculosis cases.

Regarding the health care network, Ribeirão Preto is divided into five Health Districts according to their location, namely: North, South, East, West and Central, totaling 49 establishments of Primary Health Care (PHC), which consist of five District Basic Health Units (DBHU), 18 Family Health Units (FHU) and 26 Basic Health Units (BHU)^10^.

As for the care for tuberculosis patients in the municipality, the BHU are responsible for conducting an active search for respiratory symptoms, performing sputum smear microscopy and/or x-ray request^11^. The treatment and follow-up of tuberculosis cases are carried out in specialized infectology outpatient clinics and do not occur in a decentralized manner in the municipality^11^.

The study population consisted of tuberculosis cases reported in the Tuberculosis Patient Control System (TBWeb), which consists of an online system in which municipal health managers report tuberculosis cases. Data collection was carried out with the approval of the Municipal Health Secretariat of Ribeirão Preto, together with the Municipal Tuberculosis Control Program.

As inclusion criteria, all confirmed and notified tuberculosis cases between 2006 and 2017, of patients living in Ribeirão Preto, were considered and, in the case of duplicate records, only the most current was considered. People whose diagnosis were made in another municipality were also excluded.

Tuberculosis cases were separated, in order to verify the behavior of the time series in different groups, but it is worth mentioning that the same case may be inserted in more than one group, according to the characteristic presented: general tuberculosis in the municipality (all cases), pulmonary tuberculosis, resistant tuberculosis, tuberculosis in children (under 15 years old), extrapulmonary tuberculosis and TB-HIV co-infection.

Regarding the analysis plan, it is worth noting that time series are characterized as a set of observations obtained, sequentially, over time^12^. Thus, the annual incidence rate of the tuberculosis groups and the notified cases grouped by administrative regions of the municipality were first calculated, considering the number of cases in the numerator, the population and the multiplication factor per 100,000 inhabitants in the denominator. Subsequently, the rates were converted to logarithmic notation (log10) in order to reduce data amplitude, using Microsoft Office Professional Plus 2016, through Excel^12^.

The Prais-Winsten auto-regression method was performed using the STATA software, version 14, to classify the time trend of the groups and regions as increasing, decreasing or stationary in the period under study. For cases in which the time trend was classified as increasing or decreasing, the Annual Percent Change (APC) and its respective 95% confidence intervals (95% CI) were calculated^12^.

The Interrupted Time Series (ITS) is described as the most effective technique to assess the impact of an intervention, with two parameters defining each segment of the series: level and trend^13^. The level is considered as the initial value of the series in each segment and the trend is the percentage change of the values over the period comprised by the segment^12^^-^ 13.

The purpose of this technique is to assess whether, when an intervention occurs, there is an immediate impact (change in level) and/or a progressive impact (change in trend) in the values of the series^12^^-^ 13. The software used for this analysis was also STATA version 14. The incidence rates were calculated month by month, the level was called “intervention” and the impact of the implementation of the RMT-TB was called “progressive post-intervention”.

The diagnosis of tuberculosis in Ribeirão Preto was performed through sputum smear microscopy and culture. In November 2014, following the recommendation of the Ministry of Health, the diagnosis of the disease was initiated in the priority municipalities through the RMT-TB carried out by the GeneXpert^®^ MTB/RIF^14^ system, which is an automated, simple, quick and easy-to-perform test in laboratories.

Therefore, 2014 was the cutoff point considered in the study, in order to verify whether, after the implementation of the diagnosis of tuberculosis through this new test, there was a change in the incidence of this disease in the municipality studied.

In the spatial analysis stage of the study, primarily with the objective of verifying the spatial dependence of the analyzed events, the Global Moran Index (GMI) was performed using the ArcGis software, version 10.5, based on inferential statistics, whose null hypothesis states that the event is randomly distributed in space, that is, there is no spatial dependence. If the result is statistically significant (p<0.05), the alternative hypothesis that indicates spatial dependence of the analyzed events is accepted. In numerical terms, the GMI can vary between -1 and +1, with negative values indicating the existence of inverse autocorrelation and positive values indicating direct correlation^15^.

Subsequently, the geographical coordinates (latitude and longitude) of the tuberculosis cases were obtained using the Google Earth Pro tool and the georeferencing was performed using the ArcGis software, version 10.5, the census sector of the municipality being used as a unit of analysis. The same procedure was carried out for the 49 health units in the city.

Subsequently, a point density analysis defined as a Kernel intensity estimator was performed, which consists of an exploratory interpolation method based on the definition of circular areas of influence around points of occurrence of a phenomenon, generating a density surface for the identification of vulnerable areas^16^^-^ 18.

The Kernel estimator has as its basic parameters the radius of influence, which defines the neighborhood of the point to be interpolated and controls the smoothness of the generated surface and an estimation function, with smoothing properties of the phenomenon.

Thus, the Kernel estimator is very useful to provide an overview of the distribution of the sample points, as well as indicating the occurrence of clusters^17^^-^ 18. In this way, thematic maps of the density distribution of the cases of pulmonary tuberculosis, resistant tuberculosis, tuberculosis in children, extrapulmonary tuberculosis, TB-HIV co-infection and general tuberculosis in the municipality, were generated in the ArcGIS 10.5 software.

The study was approved by the Research Ethics Committee of the Ribeirão Preto School of Nursing, with Certificate of Presentation for Ethical Appreciation (CAAE) No. 87696318.3.0000.5393 and protocol number: 3,294,221.

## Results

Between 2006 and 2017, 2,259 cases of tuberculosis were reported in Ribeirão Preto, 1,760 (77.9%) of which were pulmonary, 19 (0.8%) of resistant tuberculosis, 98 (4.3%) of tuberculosis in people under 15 (children), 497 cases (22%) of extrapulmonary type and 510 (22.6%) of TB-HIV co-infection.

The temporal trend of tuberculosis in the municipality was classified as decreasing, with a reduction of 18.1% per year (95% CI=-1.14 to -32.23) and, also, decreasing for tuberculosis in children (under 15 years old), with a 6.9% reduction per year (95% CI=-0.45 to -10.87). Regarding the forms of pulmonary, resistant, extrapulmonary tuberculosis and TB-HIV co-infection, the temporal trends were classified as stationary, as shown in Table 1.

**Table 1:** Temporal trend of the incidence of tuberculosis (n=2259). Ribeirão Preto, SP, Brazil, 2006-2017

Variables	Coefficient (95% CI)*	Trend	APC^†^ (95% CI)*
General tuberculosis	-0.087(-0.169 ; -0.005)	Decreasing	-18.1%(-1.14 ; -32.23)
Pulmonary tuberculosis	0.001(-0.005 ; 0.007)	Stationary	-
Resistant tuberculosis	0.024(-0.034 ; 0.083)	Stationary	-
Tuberculosis in children	-0.029(-0.055 ; -0.002)	Decreasing	-6.9%(-0.45 ; -10.87)
Extrapulmonary tuberculosis	0.002(-0.016 ; 0.021)	Stationary	-
TB-HIV co-infectio^‡^	-0.016(-0.041 ; 0.008)	Stationary	-

* 95% CI = 95% Confidence Interval;

† APC = Annual Percent Change;

‡ TB-HIV co-infection = Tuberculosis and Human Immunodeficiency Virus co-infection

In the grouping by administrative regions, 554 cases of tuberculosis in the North District, 441 cases in the South District, 311 in the East District, 599 in the West District and 354 cases in the Central district were reported during the study period. The temporal trend in the incidence of tuberculosis in the North District was decreasing, with a reduction of -6.67% per year (95% CI: -2.27 to -10.66) and the East District showed an upward trend of 17.4% per year (95% CI: 6.90 to 28.82). The South, West and Central Districts showed a stationary trend.

Table 3 presents the results obtained by the Interrupted Time Series technique. In the analysis, there was no change in level with the implementation of the RMT-TB in the municipality, in relation to general, pulmonary, child, extrapulmonary tuberculosis and TB-HIV co-infection; however, a change is observed in the temporal trend of resistant tuberculosis, being classified as increasing in the post-intervention period, that is, after the implementation of the RMT-TB by the GeneXpert^®^ MTB/RIF system, there was an increase 0.6% per year (95% CI=0.230 to 1.157) in the incidence of this condition, in the municipality.

**Table 2:** Temporal trend in the incidence of tuberculosis (n=2259) according to Health Districts. Ribeirão Preto, SP, Brazil, 2006-2017

Variables	Coefficient (95% CI)*	Trend	APC^†^ (95 CI)*
North	-0.03(-0.01 to -0.04)	Decreasing	-6.67%(-2.27 to -10.66)
South	-0.01(-0.03 to 0.03)	Stationary	--
East	-0.07 (0.02 to 0.11)	Increasing	17.4%(6.90 to 28.82)
West	-0.04(-0.11 to 0.03)	Stationary	--
Central	0.07(-0.08 to 0.22)	Stationary	--

* 95% CI = 95% Confidence Interval;

† APC = Annual Percent Change

**Table 3:** Application of the Interrupted Time Series to measure the impact on the detection of sensitive and resistant TB. Ribeirão Preto, SP, Brazil, 2006-2017

	INTERVENCIÓN			POSINTERVENCIÓN*		
Variables	Coefficient (95% CI)*	Trend	APC^†^ (95 CI)*	Coefficient (95% CI)*	Trend	APC^†^ (95 CI)*
General tuberculosis	-0.018 (-0.133; 0.096)	Stationary	-	0.001 (-0.003; 0.005)	Stationary	-
Pulmonary tuberculosis	-0.026 (-0.149; 0.097)	Stationary	-	0.003 (-0.001; 0.007)	Stationary	-
Tuberculosis in children	0.005 (-0.270; 0.281)	Stationary	-	-0.004 (-0.015; 0.006)	Stationary	-
Extrapulmonary tuberculosis	0.108 (-0.093; 0.309)	Stationary	-	-0.004 (-0.012; 0.003)	Stationary	-
TB-HIV co-infection^§^	-0.027 (-0.221; 0.166)	Stationary	-	-0.001 (-0.009; 0.005)	Stationary	-
Resistant tuberculosis	-0.040 (-0.090; 0.010)	Stationary	-	0.003 (0.001; 0.005)	Increasing	0.6%(0.23; 1.15)

* Post-Intervention: Beginning of the Rapid Molecular Test for Tuberculosis through the GeneXpert® MTB/RIF system in November 2014;

† 95% CI = 95% Confidence Interval;

^‡^APC = Annual Percent Change;

§ TB-HIV Coinfection = Tuberculosis and Human Immunodeficiency Virus co-infection

Regarding the spatial analysis stage, for general tuberculosis, the GMI was 0.27 (p=<0.01); pulmonary tuberculosis GMI=0.01 (p=<0.01); in children GMI=0.01 (p=<0.01); extrapulmonary GMI=0.12 (p=<0.01); TB-HIV co-infection GMI=0.16 (p=<0.01) indicating spatial dependence of the analyzed events. Only resistant tuberculosis did not show spatial dependence, with GMI=0.01 (p=0.39).

Of the total number of tuberculosis cases reported in the study period, it was possible to determine the geographic location and to georeference 2,094 (99.3%). Among the health units in the municipality, all 49 units were georeferenced.

The districts that had the highest number of tuberculosis cases were West and North, which are also the ones that have the largest number of health units (20 health units in the West District and 11 health units in the North District).

**Figure 1:**
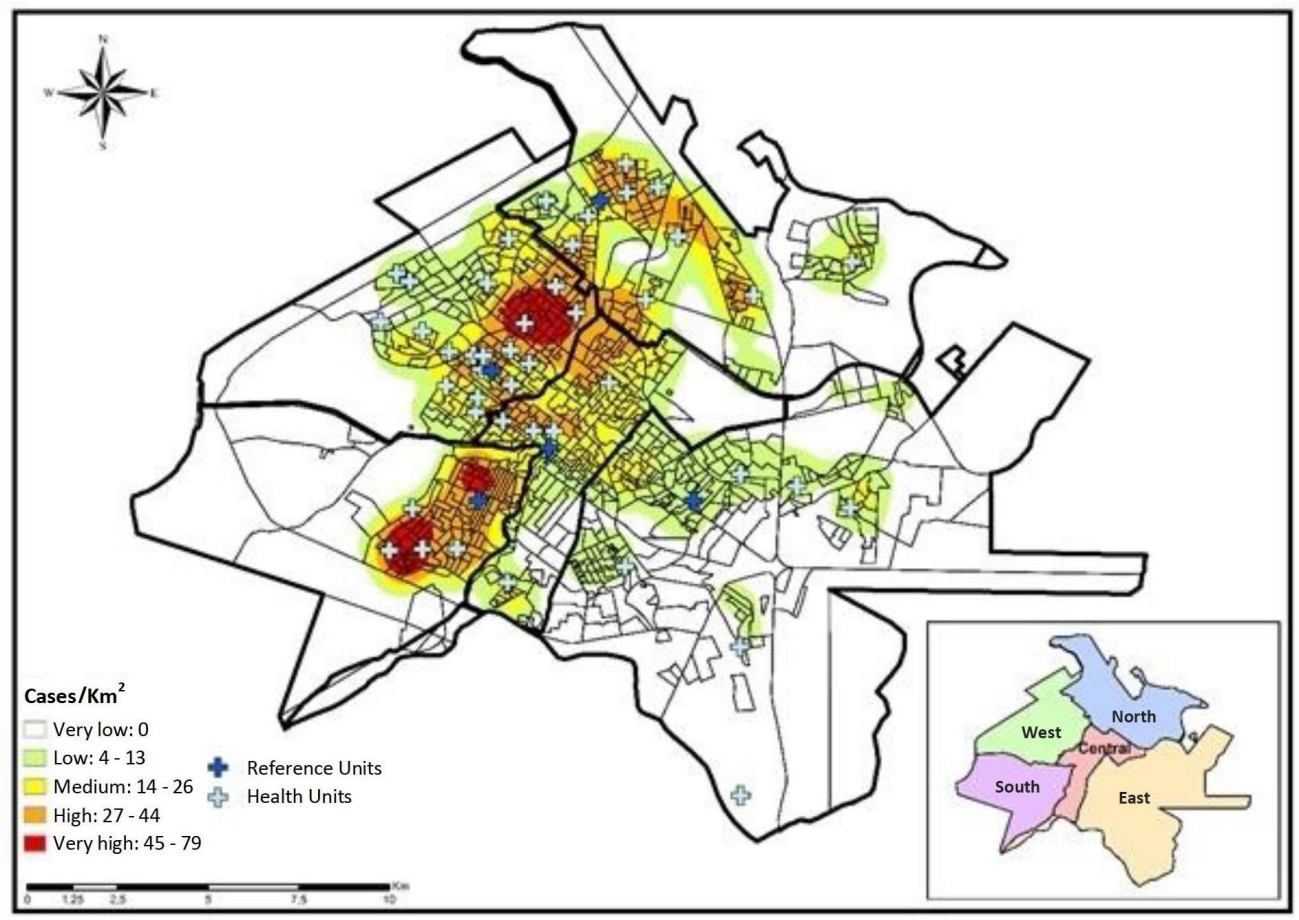
Density of tuberculosis cases (n=2094) per km^2^ and distribution of Health Units by region. Ribeirão Preto, SP, Brazil, 2006-2017

The Kernel intensity estimator allowed identifying the areas with the highest density of tuberculosis cases in the municipality, which were concentrated in the census sectors for the South and West health Districts, with a range of 45 to 79 cases of tuberculosis per km^2^, being classified as very high density and the South, West, Central and North health Districts varied from 27 to 44 cases of tuberculosis per km^2^, classified as high density (Figure 1).

**Figure 2:**
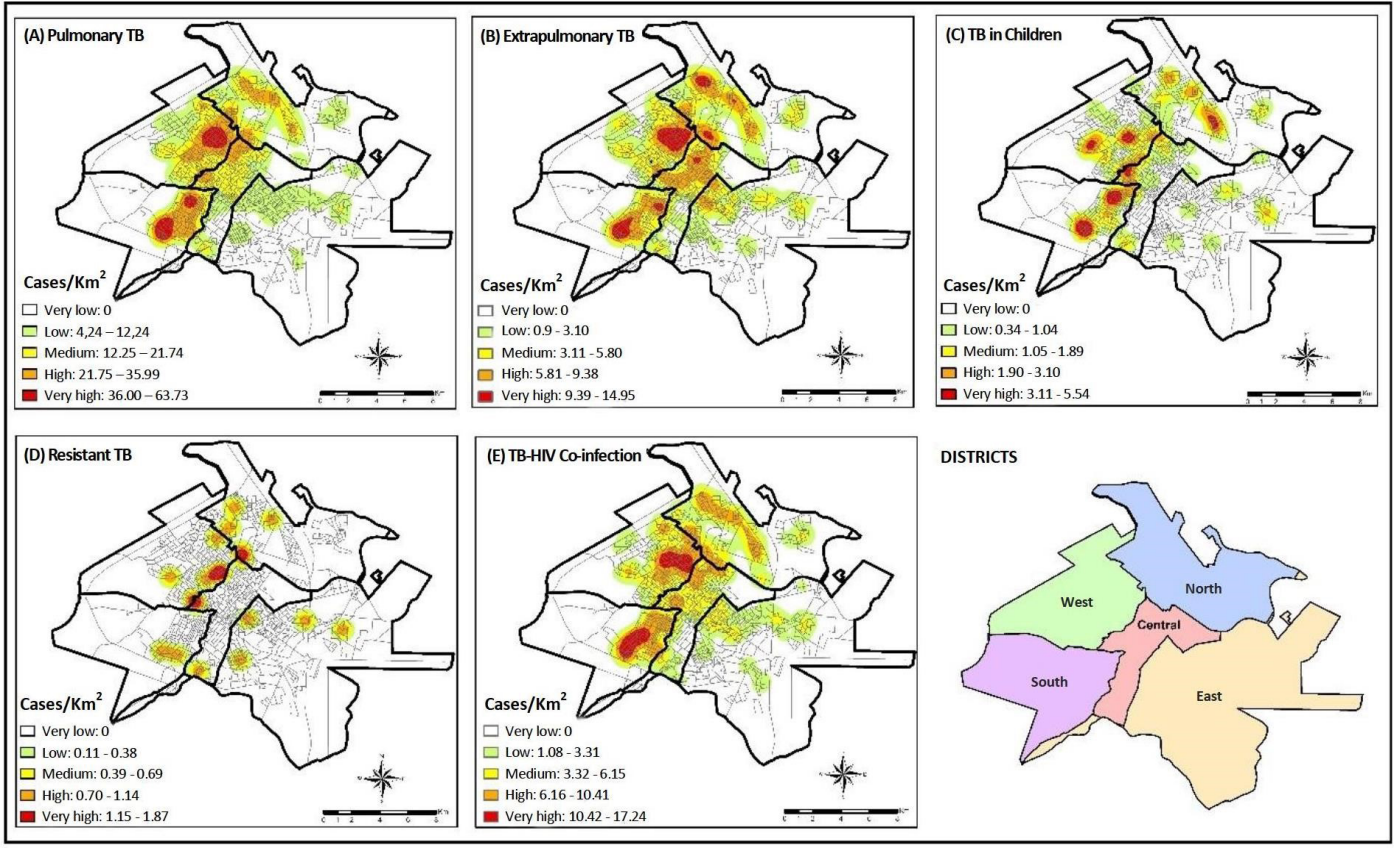
Distribution of the density of tuberculosis cases (n=2094). Ribeirão Preto, SP, Brazil, 2006-2017

As for the groups, the highest density of pulmonary tuberculosis cases was identified in the South and West Districts, varying between 36 and 63.73 cases/km^2^, as well as for TB-HIV coinfection, with a variation between 10.42 and 17.24 cases/km^2^. Regarding extrapulmonary tuberculosis, the South, West, North and Central Districts had variations of 9.39 - 14.95 cases/km^2^, being classified as very high density, as well as tuberculosis in children, with a variation of 3.11 - 5.54 cases/km^2^. Finally, resistant tuberculosis showed a higher density of cases in the Central, West and North Districts, ranging from 1.15 to 1.87 cases/km^2^, as shown in Figure 2.

## Discussion

The study aimed to assess the impact of GeneXpert^®^ MTB/RIF in the detection of tuberculosis, to analyze the temporal trend of the event, and to identify vulnerable territories in Ribeirão Preto, State of São Paulo, Brazil, a city considered a priority in the control of the disease.

The temporal trend of tuberculosis in this endemic municipality in the inland of São Paulo, was classified as decreasing. According to the World Health Organization, globally, the incidence and mortality rates due to tuberculosis are falling; however, the disease remains an important public health issue^1^. This drop in its rates can be the reflection of the strategies launched to eliminate the disease, such as the End TB Strategy, which is based on three pillars: integrated care and prevention, centered on the patient; bold policies and support systems, in addition to intensifying research and innovation^19^.

However, the decreasing trend identified must be viewed with caution as, instead of indicating that the policies and strategies to combat tuberculosis are succeeding, they can indicate that new cases are not being diagnosed and/or reported. Thus, the question arises that this decreasing trend is real or a reflection of under-detection and/or under-reporting of cases, which, in this context, is an alert for municipal epidemiological surveillance, to identify situations in which the reported data may differentiate from the true behavior of the disease^20^.

The temporal trend of tuberculosis in people under 15 years old was also classified as decreasing. It is important to note that children can only be infected after birth through close contact with an adult with tuberculosis still in the bacilliferous phase and, therefore, the diagnosis of tuberculosis in childhood is considered a sentinel event that warns against the presence of sick adults in the children’s environment^21^^-^ 23.

The big challenge related to childhood tuberculosis is its diagnosis^21^^-^ 23, hindered by the absence of an exam that can be considered as gold standard, once again, raising the question about whether the disease indices are really decreasing.

According to the results observed in another study^24^, the sensitivity of RMT-TB in children was 80%, with induced sputum when compared to culture (gold standard); even if gastric lavage is considered, sensitivity reaches 90%, with culture as the standard test. In contrast, the sensitivity of smear microscopy in children is close to zero when compared to culture; if gastric lavage is used, the sensitivity of sputum smear increases slightly, around 20%.

Another hypothesis that is raised in view of the results obtained is that, even not showing a significant result in the Interrupted Time Series analysis, this decrease in the incidence of childhood tuberculosis could be related to the greater sensitivity of the RMT-TB, since the diagnostic techniques classically used in adults have low sensitivity and specificity in children and confirmation by bacteriological identification is not always possible^21^, with the child having to start treatment without an accurate diagnosis for the disease.

Therefore, it is suggested that future studies be carried out after more use of this new diagnostic technology has elapsed, in order to find the possible relationship between the decrease in the incidence of tuberculosis in children under 15 and the greater RMT-TB sensitivity, performed by GeneXpert ^®^ MTB/RIF.

Also referring to the use of the Prais-Winsten technique to classify the temporal trend of the incidence of tuberculosis, it was identified that the East District of the municipality has an increasing trend and the North District, a decreasing trend. In Ribeirão Preto, health units actively search for respiratory symptoms according to their coverage area^11^.

Thus, health education is considered one of the most effective strategies for qualifying health workers, with a view to improving the quality of care provided to the community and early detection of new cases of tuberculosis, in addition to contributing to the organization, planning and implementation of the assistance offered to the population^25^.

In a literature review^26^, the need was identified to train health professionals so that they can respond to the demands of people with tuberculosis and develop actions of active search in the community, enabling the identification of respiratory symptoms in critical and/or vulnerable areas, so that the diagnosis can happen early. In addition, during professional training actions, prevention and health education actions should be encouraged, not only focusing on the person with tuberculosis, but also on their family and community.

Through the Interrupted Time Series technique, it was identified that, after the implementation of the RMT-TB, there was an increase in the diagnosis of resistant tuberculosis in the municipality (0.6% per year). A number of studies^4^^-^ 6 show that, in samples with negative smear microscopy, the sensitivity of the RMT-TB for a sputum sample is 72.5% and that, for three samples, it reaches almost 91%. Specificity reaches 99%. The test also detects resistance to rifampicin with 99.1% sensitivity and excludes resistance with 100% specificity.

Determined by previous study^4^, RMT-TB has high specificity in detecting resistance to rifampicin (98%). Another investigation^27^ showed the high positive predictive value for resistance to rifampicin (90.2%) in countries where the prevalence of tuberculosis is low. In addition, it also demonstrated, that 82% of the cases of resistance to rifampin diagnosed using RMT-TB were subsequently confirmed as cases of drug-resistant tuberculosis.

Based on the assumption that there can be under-detection of cases, geotechnologies can be useful tools to assist in the identification of priority areas and, thus, improve the effectiveness of control measures and, also, reduce operating costs^28^.

The GMI confirmed the spatial dependence of cases of general, pulmonary, childhood, extrapulmonary tuberculosis, and TB-HIV co-infection, corroborating the findings of other studies^29^^-^ 30. Tuberculosis is a disease strongly associated with the Social Determinants of Health, which include factors such as income, schooling, social class, race/skin-color, housing conditions, work and nutrition. Therefore, health actions with a view to its control must be of greater reach and broad spectrum under the logic of inter-sectoriality, with mobilization and participation of all sectors and of society^31^, under penalty of not reversing the reality found in the study.

Using the Kernel point density technique, it was possible to observe that the clusters do not form randomly in space, verifying that the cases of tuberculosis are unevenly distributed, in the municipality. Thus, areas with a very high density of cases of the disease were identified in the Central Districts, whose main characteristic is a high rate of homeless people, the South District, which has the largest subnormal cluster in number of residents in the municipality, and the North and West Districts, which have areas with high population density, high concentration of poverty and intermediate living conditions^32^.

The West and North Districts had the highest number of tuberculosis cases and, also, the highest number of health units. It is worth noting that the patient’s proximity to the health units does not guarantee access to the diagnosis of the disease and, consequently, to effective treatment, since access to these services can often be compromised for various reasons of a professional and/or personal character^33^.

It is noteworthy that tuberculosis is considered a neglected disease because it is, mainly, related to conditions of poverty and population clusters, in order to perpetuate a cycle of inequalities and stigma. A number of research studies suggest that the main effect of the stigma associated with tuberculosis is the social isolation of the person affected by the disease and the fear of diagnosis, which can be observed through cases that move to be treated in health units, far from their residence or neighborhood^33^^-^ 34.

Currently, studies that seek to identify the relationship between diseases and geographic space have demonstrated their importance in the scientific world, with practical applications, by means of the health service managers, although the use of geotechnologies and spatial analysis techniques is still uncommon^35^. Thus, it is of great importance to understand the dynamics of the disease in space, in order to enable the development of health surveillance actions as well as control strategies, aiming at early diagnosis and correct treatment and, thus, making it possible to break the chain of transmission.

Regarding the limitations of the study, it is worth mentioning the use of secondary data sources, which can lead to incomplete data or typos; in addition, the short time elapsed from the implementation of the diagnosis of tuberculosis through the RMT-TB.

The RMT-TB performed by the GeneXpert^®^ MTB/RIF system, as previously highlighted, is the method currently recommended by the Ministry of Health for the diagnosis of tuberculosis and its main advantage is faster and more accurate diagnosis, when compared to the other classic diagnostic methods, such as smear microscopy and culture^2^^-^ 3.

An important issue that must be brought to the study is that not all Brazilian municipalities have this technology (only 450 included in 2014), making sputum smear microscopy one of the only options and, in this sense, the study serves as an important evidence base for changing this reality. Notably, on sputum smear microscopy, despite being an undemanding test in terms of infrastructure and being low-cost, its sensitivity is 60% to 80% of the cases of pulmonary tuberculosis^3^^-^ 5, which increases the chances of underreporting, especially among paucibacillaries, such as people living with HIV and children.

Culture, which is a laboratory test considered the gold standard for the diagnosis of tuberculosis, can increase the diagnosis of the disease by 30% in cases that tested negative for smear microscopy. However, culture is little used for diagnosis, since Mycobacterium tuberculosis reproduces slowly, in around 4 to 8 weeks(3-5) and, in this way, aiming at a quick start of treatment to minimize the time of the patient in the bacilliferous phase, waiting for the culture result to start drug therapy ends up becoming unfeasible.

The main advantages of the RMT-TB performed by the GeneXpert^®^ MTB/RIF system are to provide results with agility, mainly in the Emergency Care Units and, concurrently with the positive or negative result for tuberculosis, already identify if the bacillus is resistant to rifampicin, the main drug used to treat the disease^3^^-^ 5. In addition, it can be operated in the same physical space where smear microscopy is performed and does not require special conditions for biosafety^3^^-^ 5.

Regarding the costs of this new diagnostic technology, the mean expenditure for GeneXpert^®^ MTB/RIF was R$ 35.57 (Minimum R$ 33.70 - US$ 6.53; Maximum R$ 39.40 - US$ 7.63) with main expenses related to inputs and reagents (62%). The mean cost of sputum smear microscopy was R$ 14.16 - US$ 2.74 (Minimum R$ 11.30- US$ 2.19; Maximum R$ 21.00 - US$ 4.07) with main expenses related to human resources (58%)^36^.

Two bacilloscopies are recommended by the National Tuberculosis Control Program, to achieve a sensitivity of 70% and, therefore, represent 80% of the value of a test performed by the Xpert^®^ MTB/RIF system, which has 88% sensitivity. Therefore, the Xpert^®^ MTB/RIF system is considered a technology with an accurate result, cost-effective in diagnosis and faster than the conventional tests^36^.

This study advances knowledge as it presents the impact of RMT-TB on the routine of the health service, in order to classify the temporal trend of tuberculosis, pulmonary tuberculosis, resistant tuberculosis, extrapulmonary tuberculosis, TB-HIV co-infection, and tuberculosis in children, in which a warning is raised about the downward trend found. In addition, it demonstrated the effectiveness of RMT-TB in the detection of resistant tuberculosis, being a serious public health problem; however, the importance of carrying out future studies, is emphasized after more use of this new technology has elapsed.

It is worth mentioning that, with the use of spatial analysis tools, it is possible to define priority areas for control actions in the territory and allow for the early diagnosis of the disease, since tuberculosis still persists as the main cause of death due to infectious diseases in the world, and social policies directed at the most disadvantaged groups, combined with multi-sectoral and interdisciplinary actions, are indispensable for the control of the disease.

## Conclusion

It was not possible to identify changes in the incidence of sensitive tuberculosis after the implementation of RMT-TB in the municipality. Although 3 years is a relatively short time to indicate the effect of this new technology, the evidence points to the impact of GeneXpert^®^ MTB/RIF system in the detection of resistant tuberculosis, which at first appeared not to be problematic in the scenario. However, the present study indicated an increase in the rates of this condition after the beginning of the diagnosis by means of RMT-TB, which automatically detects the resistance of the bacillus to rifampicin.

With the use of spatial analysis tools, it was possible to identify areas that must be considered a priority in the municipality, so that the managers have to prioritize these regions in actions to combat tuberculosis, with an active search for respiratory symptoms aiming at breaking the chain of bacillus transmission and at disease control.
